# Immune Biomarkers in Fibromyalgia After a Qigong Programme: An Exploratory Randomized Controlled Study

**DOI:** 10.1002/hsr2.72164

**Published:** 2026-03-22

**Authors:** Juan M. Manzaneque, Francisca M. Vera, Francisco M. Rodriguez‐Peña, Soledad Sanchez‐Montes, Maria J. Blanca

**Affiliations:** ^1^ Department of Psychobiology and Methodology of Behavioral Science University of Malaga Malaga Spain; ^2^ UGC Clinical Laboratory Regional University Hospital of Malaga Malaga Spain; ^3^ Biochemical Commission on Immunological Diseases (Semedlab) Malaga Spain

**Keywords:** fibromyalgia, immune biomarkers, mind‐body therapies, psychosomatic, qigong

## Abstract

**Background and Aims:**

Qigong is an ancient Chinese psychosomatic system with a fascinating holistic approach to health, which exerts remarkable physical and mental benefits. Nevertheless, this method has been scarcely investigated in fibromyalgia, and although a significant amount of research has focused on the immune effects of qigong, its action on immune parameters of individuals with fibromyalgia has never been studied to date. Thus, the aim of the present study was, therefore, to explore the effects of a qigong programme on white blood cells and other immune parameters in individuals with this syndrome.

**Methods:**

39 individuals participated in the study, 16 in the experimental group and 23 in the control. Experimental individuals participated in a 4‐week qigong programme. Blood samples for the quantification of immune parameters (leukocyte count, number and percentage of specific leukocyte and lymphocyte subsets, as well as concentrations of immunoglobulins and complement) were drawn from all participants before the experiment commenced and after it concluded.

**Results:**

The experimental group displayed a significantly lower value in the number of specific lymphocytes subsets such as CD3, CD4, CD8, CD16, CD45, as well as in the percentage of total lymphocytes. In addition, the experimental group exhibited a greater percentage of CD19 and a higher concentration of C3.

**Conclusion:**

The practice of qigong for a short period of 1 month was associated with significant changes of diverse immunological biomarkers in individuals with fibromyalgia. These changes were characterized by a higher number of numerous lymphocyte subsets, while at the same time a lower concentration of C3 and of the percentage of some lymphocyte subtype in these individuals. While it is tempting to speculate the implications of the broad immunomodulation associated with qigong practice in fibromyalgia syndrome, further research into the immune effects of this Taoist mind‐body practice is needed.

## 1 Introduction

Qigong is an ancient health preservation system which is part of Traditional Chinese Medicine [1, 2]. This psychosomatic discipline, of which there are several different methods, aims to achieve mind body harmony by integrating body movement, breathing, and meditation into one single multifaceted exercise [3, 4, 5]. Given the peculiar combination of elements that make up its practice, qigong has been said to be a form of moving meditation [6, 7, 8].

With its fascinating holistic approach, it is not surprising that qigong has been reported to exert numerous health benefits [9, 10, 11, 12, 13, 14]. Specifically, biomedical research has revealed that qigong can induce a broad range of physiological benefits [15, 16, 17, 18, 19, 20], as well as improvement of anxiety and depression symptoms [21, 22, 23, 24, 25, 26]. In addition, qigong has been shown to improve certain medical conditions ranging from hypertension [22, 27, 28], chronic heart disease [29, 30, 31], movement disorders [32, 33, 34], fibromyalgia [35, 36, 37], and cancer [38, 39, 40].

Fibromyalgia is a chronic pain condition estimated to affect 2% of the population [41, 42]. It is a common and debilitating disease with many symptoms that affect the quality of life [43]. Its main features are musculoskeletal pain, typically accompanied by other problems, such as fatigue, sleep disturbance and cognitive difficulties [44, 45]. Although the immune system has been suspected for some time to be implied in fibromyalgia [46, 47], the pathophysiology of the disease remains elusive, and treatment, which is typically empirical, provides limited relief [48, 49]. Thus, to ascertain the precise role of the immune system in the pathology would be a most relevant task, as not only a clearer picture of the etiology of the disease may arise from this study, but also a target for possible treatments. Besides fibromyalgia immunological background, there is evidence to support that inflammation‐driven pathways are involved in the pathogenesis of this syndrome [50].

Albeit the number of fibromyalgia studies is extensive, the number of those that have focused on the immune features of the disease is, comparatively, scarce. This is surprising given that the involvement of the immune system in the syndrome seems of such magnitude that fibromyalgia has been suggested to be an autoimmune disease [51]. Recently, however, a renewed focus with respect to the role of the immune system in generating fibromyalgia pain has been recently reported [52]. Thus, fibromyalgia has been studied in relation to immune parameters such as cytokines [53, 54, 55], antibodies [56, 57, 58], and leukocyte subpopulations [59, 60, 61].

On the other hand, the number of studies using qigong as a behavioral intervention in the pathology is very limited, in spite of the considerable therapeutic potential of this psychosomatic discipline for fibromyalgia. This remarkable potential is due to the holistic nature of the method as well as the extensive mind/body implications of this specific pathology. Likewise, there is not any single study focusing on the variations of immune parameters after qigong training in individuals with this syndrome, albeit qigong has been reported to induce changes of these parameters in healthy subjects [62, 63]. This type of study would be extremely important, since the immune response to qigong training on the part of these individuals may differ from the response to be found in healthy individuals, and the difference in this response may highlight the implication of certain immune parameters in the disease. Therefore, the main goal of the present exploratory study was the quantification and assessment of numerous immune parameters in participants with fibromyalgia after submitting these individuals to a qigong training programme for the length of 1 month.

## 2 Methods

### 2.1 Participants

Participants in this study were individuals with fibromyalgia from a local association by the name *Afibroma* in the city of Malaga, who, on a volunteer basis, were recruited to be part of the experimental or control group after a preliminary interview. They were scrutinised about their health, as well as lifestyle habits, including sleep quality, eating and drinking routines, and physical and leisure activities.

The inclusion criteria for both the experimental and control groups were as follows:

− A physician‐confirmed diagnosis of fibromyalgia based on the American College of Rheumatology 2010 criteria.
− Absence of serious medical conditions that could hinder participation in the study.
− Maintenance of regular and stable lifestyle habits.
− If taking medication for pain, sleep disturbances, or mild symptoms of anxiety or depression, individuals must be under a stable dosage.
− No involvement in sports, qigong, yoga or other similar disciplines while the study was underway and for 1 month before it started.

The exclusion criteria for would‐be participants were the following ones:

− A diagnosis of major depressive disorder, severe psychiatric illness, or active cancer.
− Having poorly controlled medical conditions, including autoimmune diseases, endocrine disorders, kidney disease, as well as cardiovascular or pulmonary illnesses.
− Taking prescribed medications other than analgesics, hypnotics, anxiolytics or antidepressants, or individuals taking these under an unsteady dosage.

All participants were requested to keep their usual lifestyle throughout the study period, so as to ensure that the only difference between the experimental and control groups was the qigong intervention followed by the former group.

The sample consisted of 42 individuals (all women), aged between 35 and 65 years. 18 were assigned to the experimental group and 24 to the control group. Of these participants, two from the experimental group and one from the control group withdrew from the study, so the group sizes were 16 and 23, respectively. Participants were randomly allocated to these two groups using a computer‐generated randomization sequence. The sequence was prepared by an independent researcher uninvolved in participant enrollment or outcome assessment, ensuring allocation concealment and minimizing selection bias. Welch *t*‐test for independent samples was performed for age, height, weight, and Body Mass Index in order to analyse the differences between both groups. The results are displayed in Table 1, showing no differences between them.

**Table 1:** Mean ± standard deviation, of age, height, weight, and body mass index (BMI) in the control and experimental groups, as well as Welch *t*‐test for independent samples and *p* value.

Variable	Control group	Experimental group	*t*	*p*
Age (yr)	55.17 ± 8.73	56.12 ± 6.54	−0.39	0.71
Height (cm)	161.39 ± 5.40	161.00 ± 5.75	0.21	0.83
Weight (kg)	67.85 ± 14.37	71.43 ± 9.83	−0.92	0.39
BMI	25.98 ± 4.96	27.62 ± 4.13	−1.12	0.27

### 2.2 Intervention

The specific method of qigong taught and practiced in this study belongs to the Taoist school of qigong, which is characterized by natural body movements, simple postures, and natural breathing [64]. It contains one single sequence of exercises, consisting of seven different movements, which are performed one after another in succession, while repeating six times each of the movements. Throughout the sequence, practitioners adopt a standard stance with their feet parallel to one another, shoulder width apart. The movements that make up the entire sequence of exercises involve the stretching of the arms, trunk, and legs, without moving the basic position of the feet and hardly changing the stance. While the movements are being performed, a natural non‐controlled breathing is required.

The qigong training programme lasted for 1 month and typically comprised three group sessions per week and some additional individual practice on the rest of the days. The group sessions involved the practice of the entire qigong form (sequence of movements) for two times, with a few minutes break in between. The training, which lasted for 30 min, was carried out on Monday, Wednesday and Friday afternoon in the premises of the fibromyalgia association (*Afibroma*), and was managed by an instructor of this qigong style. The instructor's role was to physically demonstrate the exercises so as to let participants practice the sequence by following and learning the movements correctly. Likewise, while participants were performing the exercises, the instructor would correct and adjust the positions displayed by them if these deviated from the standard ones. This type of correction by the instructor typically took place during the first week of training, when participants were still learning the movements. Besides the group sessions, individuals from the experimental group were also required to practice at home on their own, once or twice a week. For the home sessions, they were asked to practice the qigong sequence one single time, which involved 10–15 min of practice. Throughout the 4‐week qigong programme, participants in the control group were requested to maintain their usual lifestyle habits.

The qigong instructor was aware that the intervention was conducted as part of a research study but was not informed of the specific study hypotheses or outcome measures. The instructor's role was limited to delivering the intervention according to a standardized protocol, uninvolved with data collection, outcome assessment, or data analysis.

### 2.3 Blood Sampling

Blood samples for the quantification of immune parameters were drawn from all participants (experimental and control) 1 day before the qigong programme began and 1 day after the final session (4 weeks later). Blood was collected by venepuncture at 9.00 a.m. and was immediately centrifuged. Samples were transported in isothermal fridges, with controlled temperature, using dry ice. Once in the laboratory, those parameters needing immediate processing (blood counts) were processed accordingly. For the rest of the biological measures, serum samples were refrigerated at 4°C–8°C, and processed within 24–48 h. The immune parameters considered included the total number of leukocytes, the number and percentage of neutrophils, monocytes, eosinophils, basophils, and lymphocytes, as well as the number and percentage of specific lymphocyte subsets such as CD3, CD4, CD8, CD16, CD19, CD45, including the CD4/CD8 ratio. Likewise, the concentrations of IgA, IgG, IgM, IgE, C3, and C4 were also measured. For the white series count (total leukocytes, monocytes, neutrophils, eosinophils, basophils, total lymphocytes, and lymphocyte subsets), the procedure involved placing 5 ml of whole blood into EDTA tubes and analysing the samples by means of XN‐9100 Hematology analyser (Sysmex). This analyser combines flow cytometry with an automatic dyer‐extensor using standard peroxidase dyeing of reference for leukocyte differential count. The concentrations of immunoglobulins and complement factors were analysed by an immunoturbidimetric method whereby complement anti‐proteins and anti‐immunoglobulin antibodies react with the antigen of the sample forming an antigen‐antibody complex. Subsequent agglutination in both cases may then be measured by turbidimetry. All the parameter analyses were performed using the Atellica Solution Immunoassay & Clinical Chemistry Analyzers (Siemens Healthineers). Reference values were calculated by the laboratory itself, considering its population. The reference value includes 95% of the population values, which will be considered normal for the parameters measured.

### 2.4 Data Analysis

Data were collected according to a pretest‐posttest design with random assignment to the control and experimental groups. First, descriptive statistics were calculated for pretest and posttest values for each group, including the difference between both measurements (Δ). Second, given the exploratory nature of the study and in line with the design, a between‐group analysis of covariance (ANCOVA) was performed on the dependent variables listed in Table 2 to explore potential post‐intervention differences between groups. The qigong programme was considered as an independent variable with two levels (absence or control group, and presence or experimental group), while the respective pretest scores of each dependent variable were treated as covariates. Thus, the posttest differences between groups were estimated with the differences in pretest scores removed, with a significance level of 0.05. All analysis fulfilled the assumptions of homogeneity of variance and homogeneity of regression slopes. Partial eta‐squared was computed as a measure of effect size. Following Cohen's criterion [65], a value of 0.01 was considered as small effect size, 0.06 as medium, and 0.14 as large.

**Table 2:** Means and standard deviation (in parentheses) in the pretest and posttest for the control and experimental groups, as well as the differences between both means (Δ).

	Control group			Experimental group		
Variables	Pretest	Posttest	Δ	Pretest	Posttest	Δ
Leukocytes	6.95 (1.47)	6.77 (1.74)	−0.18	6.63 (1.76)	7.14 (2.18)	0.51
Neutrophils	3.53 (1.05)	3.25 (1.08)	−0.28	3.38 (1.13)	3.82 (1.85)	0.44
Neutrophils %	50.50 (8.76)	47.63 (6.93)	−2.87	50.40 (7.31)	51.95 (9.11)	1.55
Lymphocytes	2.67 (0.80)	2.74 (0.80)	0.08	2.48 (0.71)	2.48 (0.70)	−0.01
Lymphocytes %	38.50 (8.52)	40.67 (6.70)	2.18	37.88 (7.28)	35.97 (8.28)	−1.91
Monocytes	0.53 (0.10)	0.55 (0.13)	0.02	0.54 (0.17)	0.61 (0.19)	0.07
Monocytes %	7.82 (1.56)	8.30 (1.67)	0.47	8.24 (2.08)	8.63 (2.06)	0.39
Eosinophils	0.15 (0.11)	0.16 (0.08)	0.01	0.15 (0.07)	0.15 (0.08)	0.00
Eosinophils %	2.14 (1.23)	2.37 (1.15)	0.23	2.36 (1.04)	2.35 (1.11)	−0.01
Basophils	0.05 (0.02)	0.05 (0.02)	0.00	0.06 (0.03)	0.06 (0.03)	0.00
Basophils %	0.77 (0.26)	0.83 (0.33)	0.06	0.93 (0.32)	0.88 (0.32)	−0.05
IgA	184.14 (64.15)	185.55 (65.44)	1.41	198.37 (86.97)	197.75 (84.65)	−0.62
IgG	879.09 (183.53)	898.65 (174.66)	19.57	1019.56 (235.52)	1003.19 (230.26)	−16.38
IgM	110.22 (51.58)	112.30 (52.80)	2.09	115.00 (58.76)	114.87 (59.79)	−0.13
IgE	25.14 (32.37)	23.52 (30.50)	−1.62	43.07 (57.90)	41.57 (54.48)	−1.50
C3	113.65 (23.32)	105.25 (22.89)	−8.40	103.36 (21.01)	110.04 (19.86)	6.68
C4	35.21 (11.11)	33.71 (11.70)	−1.50	31.51 (7.76)	31.06 (6.91)	−0.45
Lymphocytes CD3	1912.41 (540.93)	1919.50 (549.17)	7.09	1837.88 (659.36)	1636.31 (481.19)	−201.56
Lymphocytes CD3 %	73.30 (6.77)	71.60 (6.72)	−1.70	75.36 (4.31)	74.06 (4.55)	−1.31
Lymphocytes CD4	1339.14 (470.70)	1331.64 (453.69)	−7.50	1302.75 (551.97)	1151.69 (401.02)	−151.06
Lymphocytes CD4 %	50.75 (7.87)	49.37 (8.51)	−1.38	52.45 (7.55)	51.57 (7.21)	−0.88
Lymphocytes CD8	567.86 (211.52)	600.55 (245.25)	32.70	517.09 (193.51)	469.87 (168.71)	−47.22
Lymphocytes CD8 %	21.23 (5.80)	21.34 (5.35)	0.11	21.99 (5.87)	21.81 (6.55)	−0.18
CD4 CD8 Ratio	2.64 (1.04)	2.54 (1.12)	−0.10	2.62 (1.00)	2.66 (1.09)	0.04
Lymphocytes CD19	339.35 (179.37)	340.83 (203.07)	1.48	242.69 (117.41)	218.09 (112.97)	−24.60
Lymphocytes CD19 %	12.30 (5.26)	11.84 (5.38)	−0.46	9.71 (3.14)	10.73 (3.56)	1.02
Lymphocytes CD16	342.07 (178.40)	392.81 (166.93)	50.74	309.92 (85.08)	297.58 (98.28)	−12.34
Lymphocytes CD16 %	13.12 (5.97)	14.86 (5.87)	1.74	13.71 (4.04)	13.94 (4.26)	0.23
Lymphocytes CD45	2707.57 (841.75)	2743.00 (916.65)	35.44	2505.38 (746.67)	2240.53 (599.56)	−264.85

Eight participants showed a missing value in at least one of the dependent variables. However, the variables involved had only one missing value (2.6% = 1/39) except IgE which showed 3 values (7.7% = 3/39). In this case, the Little's test of missing completely at random did not reach statistical significance, *χ*2 (3) = 2.07, *p* = 0.56, indicating that missing values were randomly distributed across observations. Therefore, we proceeded with the analysis using completed cases for each variable. Statistical analysis was carried out by IBM‐SPSS 28.

## 3 Results

**Figure 1:**
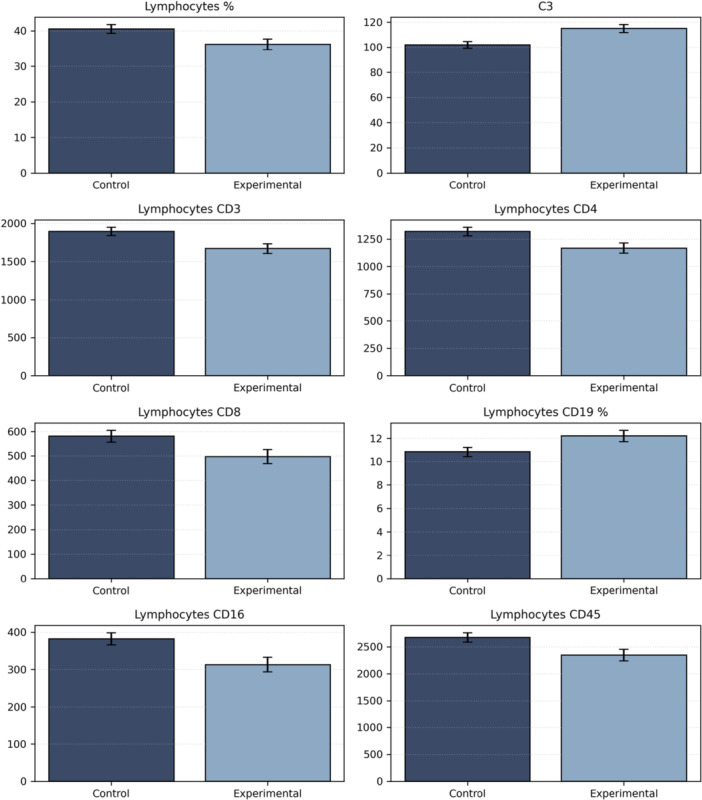
Adjusted posttest means (controlling for pretest values) with standard error for the control and experimental groups of the statistically significant variables derived from ANCOVA. ANCOVA, analysis of covariance.

Descriptive statistics for pretest and posttest for each group, as well as the results of the ANCOVA are shown in Tables 2 and 3, respectively. The ANCOVA revealed that the adjusted posttest means differ between the experimental and control groups with respect to the number or percentage of specific lymphocyte subsets, as well as the concentration of C3. Thus, the experimental group showed a lower mean in the count of several lymphocyte subsets (CD3, CD4, CD8, CD16, CD45), as well as in the percentage of total lymphocytes, as compared to controls. Likewise, the experimental group also exhibited a significantly higher mean in the percentage of CD19, and in the concentration of C3. Figure 1 shows the adjusted posttest means for the control and experimental groups of these statistically significant variables. All partial eta‐squared were close to a large effect size. Statistical significance was not reached, however, in the concentrations of IgA, IgG, IgM, IgE, and C4, in the number and percentage of neutrophils, monocytes, eosinophils, and basophils, as well as in the number of total leukocytes and lymphocytes, or the percentage of specific lymphocyte subsets.

**Table 3:** Adjusted mean of each dependent variable for control and experimental groups, standard error in parenthesis, *F* statistics, *p* value and partial eta‐squared.

Variables	Control group	Experimental group	*F*	*p*	*Partial η* ^2^
Leukocytes	6.68 (0.32)	7.28 (0.39)	1.40	0.25	0.04
Neutrophils	3.22 (0.29)	3.86 (0.35)	1.96	0.17	0.05
Neutrophils %	47.61 (1.45)	51.97 (1.79)	3.60	0.07	0.09
Lymphocytes	2.68 (0.06)	2.57 (0.08)	1.07	0.31	0.03
Lymphocytes %	40.54 (1.23)	36.18 (1.52)	4.96*	0.03	0.12
Monocytes	0.55 (0.02)	0.60 (0.03)	2.58	0.12	0.07
Monocytes %	8.43 (0.23)	8.43 (0.29)	0.01	0.99	< 0.01
Eosinophils	0.16 (0.01)	0.15 (0.01)	0.07	0.80	< 0.01
Eosinophils %	2.43 (0.18)	2.26 (0.23)	0.32	0.58	< 0.01
Basophils	0.06 (0.01)	0.06 (0.01)	0.13	0.72	< 0.01
Basophils %	0.88 (0.05)	0.80 (0.06)	1.02	0.32	0.03
IgA	191.40 (3.01)	189.70 (3.52)	0.13	0.72	< 0.01
IgG	951.75 (12.88)	926.86 (15.60)	1.44	0.24	0.04
IgM	114.19 (2.34)	111.97 (2.89)	0.36	0.55	0.01
IgE	30.48 (1.34)	31.826 (1.60)	0.41	0.52	0.01
C3	101.85 (2.58)	114.92 (3.11)	10.26**	0.003	0.22
C4	32.25 (0.70)	33.16 (0.84)	0.67	0.42	0.02
Lymphocytes CD3	1895.30 (54.38)	1669.59 (63.79)	7.24**	0.01	0.17
Lymphocytes CD3 %	72.36 (0.65)	73.02 (0.76)	0.43	0.52	0.01
Lymphocytes CD4	1319.72 (38.80)	1168.07 (45.50)	6.43*	0.02	0.16
Lymphocytes CD4 %	50.02 (0.85)	50.69 (0.99)	0.27	0.61	< 0.01
Lymphocytes CD8	581.14 (24.30)	496.57 (28.53)	5.06*	0.03	0.13
Lymphocytes CD8 %	21.63 (0.56)	21.41 (0.65)	0.06	0.80	< 0.01
CD4 CD8 Ratio	2.53 (0.09)	2.67 (0.10)	0.94	0.34	0.03
Lymphocytes CD19	305.71 (21.89)	268.56 (26.46)	1.12	0.30	0.03
Lymphocytes CD19 %	10.82 (0.41)	12.20 (0.49)	4.49*	0.04	0.11
Lymphocytes CD16	381.99 (16.11)	313.12 (19.35)	7.44**	0.01	0.17
Lymphocytes CD16 %	15.07 (0.53)	13.63 (0.63)	3.05	0.09	0.08
Lymphocytes CD45	2673.53 (85.09)	2347.05 (105.54)	5.76*	0.02	0.14

** *p* ≤ 0.01

* *p* ≤ 0.05.

## 4 Discussion

The main result of this research is that the practice of qigong in individuals with fibromialgia, for a short period of 1 month, was associated with a noteworthy and broad immunomodulation, which included changes of lymphocyte count and percentage, as well as variations in the concentration of complement C3. Likewise, it is interesting to note that, albeit qigong has already been reported to be related to changes of immune parameters in healthy individuals, this is the first time that these type of changes have been observed in a sample of individuals with fibromyalgia, and the first time, too, that the specific method of Taoist qigong has been investigated in this syndrome.

The lower value in the count of different lymphocyte subsets (CD3, CD4, CD8, CD16, CD45), as well as in the percentage of total lymphocytes, is a most significant and consistent result of our investigation, which by itself illustrates the wide immunomodulation associated with qigong practice in individuals with fibromyalgia. Unlike this consistent result, however, the total number of lymphocytes did not reach statistical significance, as did not the percentage of the above mentioned lymphocyte subsets. Likewise, in contrast to the ample downregulation described above for the number of specific lymphocytes subsets, the percentage of CD19, however, was greater in the experimental group than in control, therefore revealing this specific parameter a differential and upward trend as compared to that shown for the majority of lymphocyte measures.

The lower count in the number of various lymphocytes subsets is indeed the main result of this investigation, as it is the first time this has been described in fibromyalgia individuals after following a qigong programme. Our finding may hold relevance given the consistency of its trend and the numerous lymphocyte parameters associated to changes. What remains to be elucidated, however, is why or how the differences in the number of several lymphocyte subsets in our study was not accompanied by differences in the percentage of the same lymphocytes. At any rate, the lower value of these parameters suggests interesting implications with respect to the very nature of fibromyalgia syndrome. In line with this, a previous study of our group [66] reported a significant improvement in psychological adjustment and pain reduction in fibromyalgia individuals after following a qigong training program. However, the specific link between the above‐mentioned differences in lymphocytes subsets and the psychological improvement found in these individuals remains elusive at this point.

Although fibromyalgia has been suggested to be an immune‐mediated disease, and some studies have focused on the role played by antibodies and cytokines in the syndrome [67], very few of them have been carried out in relation to the role played by specific lymphocyte subsets [61]. Notwithstanding, fibromyalgia has been said to be related to an immune dysregulation affecting T‐cells [68]. In fact, CD8 + T cells have been reported to be involved in the occurrence and development of the syndrome [69]. In this context, our finding that the number of numerous lymphocyte subsets decreased after engaging participants in qigong practice is a novel and significant result which may lend itself to some clinical implications.

An interesting finding of the present research is the fact that the total number of lymphocytes remained unaffected in the experimental group while its percentage was significantly lower in this group than in control. These results, as a matter of fact, contrast with what was reported in a previous study by our group [1], using the same method of qigong and a very similar program. Interestingly, in our previous study, no differences were found in the percentage of total lymphocytes, which has been reported to be significantly lower in the present study, and the opposite ocurred with the total number of lymphocytes between both studies, being significantly lower in the previous study but non‐significant in the present one. Because of the many similarities between the two programs, we must conclude that the lack of concordance between both studies in relation to these two immune parameters may be due to the peculiar characteristics of the sample from the present investigation, being fibromyalgia individuals. In this regard, our present results would, therefore, highlight these two particular immune parameters, given that after qigong practice, the total number of lymphocytes was not associated with changes in individuals with fibromyalgia, unlike what was found in healthy participants, whereas the total percentage of lymphocytes was linked to changes in fibromyalgia but not in healthy individuals.

In the present research, although statistical significance was not reached in the concentration of complement C4, the C3 fraction was found to be significantly higher in the experimental group than in control. This higher value of C3 seems an interesting result that differs from what was reported in the previous study of our group, using the same methodology [1], we referred to above. Thus, employing the same type of qigong and the same programme in terms of length, days of practice per week, etc., no difference was reported in the concentration of C3 in our previous study. Once again, this entails a different response to qigong practice with respect to this immune parameter between healthy individuals and those with fibromyalgia, which lends itself to some speculation in relation to the possible role of C3 in fibromyalgia. In this regard, although C3, as of yet, has not been reported to be different in fibromyalgia patients as compared to healthy people [70, 71], the levels of C3 (and C4) have been said to be lower in individuals with anxiety and depression [72]. Since these are some of the psychosomatic symptoms of fibromyalgia [73, 74, 75], our result of a higher concentration of the C3 fraction of complement in the experimental group might be compatible with an anti‐anxiety and anti‐depressive action of qigong in these individuals.

The fact that the experimental group displayed a higher concentration of the C3 fraction of complement, while at the same time displaying reduced lymphocyte subsets is an interesting result from our research, as it lends itself to speculation with respect to the mechanism underlying this negative correlation. Thus, while the complement system has been said to be of vital importance for the differentiation and function of specific lymphocyte populations such as T cells [76], the relationship between the complement system and T lymphocytes in particular, is not well studied [77]. Notwithstanding, a negative correlation of complement C3 with some T cells has been reported under specific medical conditions [78]. Therefore, the negative correlation found in our study between these two parameters, namely, the C3 concentration and the number of various T lymphocytes, seems plausible and consistent. What remains well beyond the scope of this research, notwithstanding, is the biological mechanism underlying this specific correlation, as well as its possible implication for fibromyalgia syndrome.

This study should be regarded as exploratory. Thus, although the findings provide preliminary evidence of immunological changes associated to qigong practice in individuals with fibromyalgia, several limitations must be acknowledged. First, the small sample size limits the generalizability of the results, and may have reduced statistical power, potentially preventing some variables from reaching statistical significance. In addition, multiple ANCOVAs were conducted across a wide range of immune parameters relative to the sample size, with no correction for multiple comparisons, which may increase the risk of Type I error. Second, the relatively short duration of the qigong intervention (1 month) and the assessment of immune parameters at only two time points constitute additional limitations, as they restrict the ability to examine changes over time and the stability of the observed effects. Finally, other potential confounding variables, such as age, diet, and other lifestyle factors, were not strictly controlled and may have influenced the results. Therefore, although our results provide interesting preliminary insights, future studies should recruit larger samples, implement longer intervention periods, include more than two assessment points (e.g., incorporating intermediate measurements), and apply appropriate statistical procedures to control for multiple testing. In addition, stricter control of confounding variables—through detailed interviews, participant diaries, or similar strategies—would strengthen the validity of the findings. Overall, these improvements would provide a broader and more consistent understanding of the effects of qigong on immune parameters in individuals with fibromyalgia.

## 5 Conclusions

The practice of qigong for a short period of only 1 month was associated with a broad effect on immune parameters in individuals with fibromyalgia, by revealing numerous differences, most notably and consistently, in the count or percentage of several lymphocyte subsets. Given the characteristics of the sample, this specific immunomodulatory profile displayed by qigong may have potential clinical implications with respect to the very nature of the complex syndrome itself. Further research would be needed to clarify the full spectrum of effects that qigong may exert over immune parameters in individuals with this syndrome, the mechanisms underlying these effects, as well as the clinical relevance of these findings within the context of fibromyalgia.

## Author Contributions

**Juan M. Manzaneque:** conceptualization, data curation, formal analysis, investigation, methodology, supervision, validation, writing – original draft, writing – review and editing. **Francisca M. Vera:** conceptualization, data curation, formal analysis, investigation, methodology, supervision, validation, writing – original draft, writing – review and editing. **Francisco M. Rodriguez‐Peña:** conceptualization, data curation, formal analysis, investigation, methodology, resources, supervision, validation, writing – review and editing. **Soledad Sanchez‐Montes:** conceptualization, data curation, formal analysis, investigation, methodology, resources, supervision, validation, writing – review and editing. **Maria J. Blanca:** conceptualization, data curation, formal analysis, investigation, methodology, resources, software, supervision, writing – review and editing.

## Funding

The authors received no specific funding for this work.

## Ethics Statement

The present work has been conducted with the approval of the Ethics Committee of the University of Malaga as of October 29, 2023 (Registration number: 82‐2023‐H). Likewise, all procedures were carried out in accordance with the 1964 Helsinki Declaration and its later amendments. The nature of the study and the procedure to be followed was thoroughly explained to all participants, and written informed consent was obtained from all of them. Fellow participants did not receive any payment or compensation for their participation.

## Conflicts of Interest

The authors declare no conflicts of interest.

## Transparency Statement

The lead author Juan M. Manzaneque affirms that this manuscript is an honest, accurate, and transparent account of the study being reported; that no important aspects of the study have been omitted; and that any discrepancies from the study as planned (and, if relevant, registered) have been explained.

## Data Availability

The data that support the findings of this study are available from the corresponding author upon reasonable request.
